# Inhibition of miR-302 Suppresses Hypoxia-Reoxygenation-Induced H9c2 Cardiomyocyte Death by Regulating Mcl-1 Expression

**DOI:** 10.1155/2017/7968905

**Published:** 2017-04-11

**Authors:** Yao-Ching Fang, Chi-Hsiao Yeh

**Affiliations:** ^1^Department of Thoracic and Cardiovascular Surgery, Chang Gung Memorial Hospital, Keelung, Taiwan; ^2^College of Medicine, Chang Gung University, Taoyuan, Taiwan

## Abstract

MicroRNAs play important roles in cell proliferation, differentiation, and apoptosis, and their expression influences cardiomyocyte apoptosis resulting from ischemia-induced myocardial infarction. Here, we determined the role of miR expression in cardiomyocyte apoptosis during hypoxia and reoxygenation. The rat cardiomyocyte cell line H9c2 was incubated for 3 h in normal or hypoxia medium, followed by reoxygenation for 24 h and transfection with a miR-302 mimic or antagomir. The effect of miR-302 on myeloid leukemia cell-differentiation protein-1 (Mcl-1) expression was determined by western blot, real-time polymerase chain reaction, and luciferase reporter assays, with cell viability assays. We observed that miR-302 expression was elevated by hypoxia/reoxygenation injury and increased further or decreased by transfection of the miR-302 mimic or miR-302 antagomir, respectively. Additionally, elevated miR-302 levels increased apoptosis-related protein levels and cardiomyocyte apoptosis, and luciferase reporter assays revealed miR-302 binding to the Mcl-1 mRNA 3′ untranslated region. Our findings suggested that miR-302 overexpression aggravated hypoxia/reoxygenation-mediated cardiomyocyte apoptosis by inhibiting antiapoptotic Mcl-1 expression, thereby activating proapoptotic molecules. Furthermore, results indicating cardiomyocyte rescue from hypoxia/reoxygenation injury following treatment with miR-302 antagomir suggested that miR-302 inhibition might constitute a therapeutic strategy for protection against cardiomyocyte apoptosis during hypoxia/reoxygenation injury.

## 1. Introduction

MicroRNAs (miRs) are a group of noncoding RNAs (~20–25 nucleotides in length) that downregulate mRNA expression through binding to their 3′ untranslated region (3′UTR) [1]. Over 1000 miRs have been discovered in human beings [2], and several mechanisms of miR-induced target suppression have been described [3]. Moreover, miRs regulate cell proliferation, differentiation, apoptosis, autophagy, and development by upregulating or downregulating mRNA expression [4–8].

Cardiomyocyte apoptosis occurs when cardiac tissue is exposed to a stressor, such as ischemia and/or reperfusion, during myocardial infarction, which is a major cause of morbidity and mortality worldwide [9]. Our previous study revealed that upon cardiomyocyte hypoxia/reoxygenation (H/R) injury, alterations in miR expression occur, causing disturbances in downstream mRNA expression and apoptotic pathway regulation [10]. Other studies reported that certain diseases, such as myocardial infarction, ischemia-reperfusion, and arrhythmia, can be treated or prevented by pharmacological (mimics or antagomirs) or genetic approaches to alter miR expression [2, 11–15]. miRs can also regulate mRNA expression to mitigate H/R injury. Cheng et al. [2] reported that miR-21 inhibits cell death under H/R conditions by regulating expression of the programmed cell death 4 (*PDCD4*) gene, which is also targeted by miR-499 [2]. Additionally, they found that miR-499 mitigates lipopolysaccharide-induced cardiac cell death by inhibiting the translation of *PDCD4* and *sex-determining region* Y- (SRY-) box *6* mRNA [15]. miR-20a also inhibits expression of the apoptotic factor Egl nine homolog 3 to protect cardiomyocytes from H/R injury [11]. Furthermore, several miRs, including miR-210 (which regulates angiogenesis) [14], miR-199a (which modulates hypoxia-inducible factor-1*α* (HIF-1*α*) expression) [16], and miR-494 (which upregulates p-Akt, HIF-1*α*, and heme oxygenase-1 expression) [17], protect cells from hypoxia- or ischemia-induced damage.

During cardiomyocyte H/R injury, pro- and antiapoptotic proteins of the B-cell lymphoma 2 (Bcl-2) family are in disequilibrium in the mitochondrial membrane, leading to changes in membrane potential, leakage of cytochrome *c* from the mitochondrion, and subsequent activation and binding of caspase-9 to form apoptosomes, which ultimately activate caspase-3 and induce apoptosis [18]. The binding of myeloid leukemia cell-differentiation protein-1 (Mcl-1), an antiapoptotic protein of the Bcl-2 family, to proapoptotic proteins, such as Bas, Bid, or Bak, inhibits apoptosis [19]. miRs regulate gene expression posttranscriptionally by direct endonucleolytic cleavage or slicer-independent decay of mRNAs and posttranslationally by decreasing the rate of translation [20]. The binding of the seed sequence in miRs to a complementary sequence in the mRNA 3′UTR promotes mRNA degradation, whereas failure to bind due to the sequences not being complementary can result in translation inhibition [21]. miR-302 transcriptionally regulates gene expression and methylation [22] and supports reprogramming in stem cells during hypoxia [23, 24]. As a putative upstream regulator of Mcl-1 expression, the role of miR-302 in protecting cardiomyocytes from H/R remains unknown. Here, we investigated whether miR-302 binds to the 3′UTR of Mcl-1 mRNA and the effects of that binding activity on protecting H9c2 cardiomyocytes from H/R injury.

## 2. Materials and Methods

### 2.1. Cell Culture

The rat cardiomyocyte cell line H9c2 was cultured in Dulbecco's modified Eagle medium (Thermo Fisher Scientific, Waltham, MA, USA) containing 10% fetal bovine serum at 37°C in a humidified atmosphere containing 95% air and 5% CO_2_. Cells were rendered quiescent by serum starvation for 24 h before all experiments. H/R injury was induced by hypoxia for 3 h (i.e., incubation in oxygen and glucose deprivation medium containing 2.3 mM CaCl_2_, 5.6 mM KCl, 154 mM NaCl, 5 mM Hepes, and 3.6 mM NaHCO_3_ (pH 7.4) and under an atmosphere of 5% CO_2_, 85% N_2_, 10% H_2_, and <0.1% O_2_), followed by the addition of glucose (4500 mg/L) to the medium and reoxygenation for 24 h in a humidified atmosphere (95% air and 5% CO_2_) at 37°C. Control cells were incubated at 37°C under 95% air and 5% CO_2_ for 27 h. To upregulate and downregulate miR-302 expression, an miR-302 mimic (m302; 50 or 100 nM; Thermo Fisher Scientific) or antagomir (i302; 50–100 nM; Thermo Fisher Scientific) was transfected into cells using Lipofectamine 2000 (Invitrogen, Carlsbad, CA, USA) according to manufacturer instructions. Nonsense control (NC) miR (100 nM) was also used as an internal control. The sequences of the nonsense miR, miR-302 mimic, and miR-302 antagomir were as follows: NC, 5′-UCACAACCUCCUAGAAAGAGUAGA-3′; miR-302 mimic, 5′-UAAGUGCUUCCAUGUUUUGGUGA-3′; and miR-302 antagomir, 5′-AUUCACGAAGGUACAAAACCACU-3′.

### 2.2. Luciferase Reporter Assay

The Mcl-1 3′UTR sequences (three repeats of 5′-TCTCTAAGGACCTAAAAGCACTTT-3′) were synthesized and subcloned into a PsiCheck2 vector (psi-Mcl-1; Promega, Madison, WI, USA). The PsiCheck2 vector without the Mcl-1 3′UTR was used as a control. Cells were cotransfected with 200 ng of plasmids containing 3′UTR or nonsense sequences and 100 nM miR mimic or a 100 nM NC miR (Thermo Fisher Scientific) according to manufacturer instructions using Lipofectamine 2000 reagent (Thermo Fisher Scientific). Experiments were performed in triplicate. After 48 h, cells were harvested and their luciferase activities were measured using the Luciferase dual-reporter kit (Promega) according to manufacturer instructions.

### 2.3. Real-Time Quantitative Polymerase Chain Reaction (qPCR)

The expression of Mcl-1 and miR-302 was examined by real-time qPCR using glyceraldehyde-3-phosphate dehydrogenase (GAPDH) or U6 as internal controls, respectively. A poly-A tail was added to the extracted total RNA, which was then reverse transcribed into cDNA, to extend RNA length. Mcl-1 and miR-302 expression was quantified following cDNA annealing using the following real-time PCR primers: Mcl-1 forward, 5′-TGCTTCGGAAACTGGACATTAAA-3′ and Mcl-1 reverse, 5′-ATGGGTCATCACTCGAGAAAAAG-3′; miR-302 forward, 5′-TAAGTGCTTCCATGTTTTGGTGA-3′ and miR-302 reverse, 5′-GAACATGTCTGCGTATCTCAGACTTC-3′; GAPDH forward, 5′-ATGACTCTACCCACGGCAAG-3′ and GAPDH reverse, 5′-GGAAGATGGTGATGGGTTTC-3′; U6 forward, 5′-GCTTCGGCAGCACATATA-3′ and U6 reverse, 5′-AACGCTTCACGAATTTGCGT-3′.

### 2.4. Western Blot Analysis

Proteins were resolved based on molecular weight via electrophoresis on 8%, 10%, and 12% polyacrylamide gels, followed by transfer to a polyvinylidene difluoride membrane. The membrane was blocked and incubated with antibodies for cleaved caspase-3, cleaved caspase-9, poly ADP ribose polymerase (PARP; Cell Signaling Technology, Danvers, MA, USA), *β*-actin (Sigma-Aldrich, St. Louis, MO, USA), and Mcl-1 (Abcam, Cambridge, MA, USA). Protein levels were analyzed using an enhanced chemiluminescence detection kit (GE Healthcare, Wauwatosa, WI, USA).

### 2.5. 3-(4,5-Dimethylthiazol-2-yl)-2,5-diphenyltetrazolium Bromide (MTT)

Following incubation and treatment with different miRs under control or H/R conditions for 27 h, cell viability was tested by incubation with MTT reagent (Sigma-Aldrich) at a final concentration of 0.5 mg/mL and a temperature of 37°C for 4 h. The optical density of the purple MTT formazan product was measured at 570 nm using an enzyme-linked immunosorbent assay (ELISA) reader. The absorbance of cells transfected with NC was regarded as indicating 100% viability.

### 2.6. Lactate Dehydrogenase (LDH) Release Assay

H9c2 cells were incubated in 96-well plates under hypoxic conditions, followed by reoxygenation, and LDH released to the medium from damaged cells was measured using an LDH cytotoxicity kit (Roche, Indianapolis, IN, USA) and ELISA reader to measure the change in absorbance at 490 nm. The absorbance of cells treated with Triton X-100 (Sigma-Aldrich) was regarded as indicating 100% cytotoxicity.

### 2.7. Flow Cytometry

H9c2 cells (1 × 10^6^) were collected, washed with cold phosphate buffer, and resuspended with 1x binding buffer containing Annexin V-FITC and propidium iodide (PI; Sigma-Aldrich) for double staining according to manufacturer instructions (BD Bioscience, San Jose, CA, USA). The collected samples were analyzed by flow cytometry (Beckman Coulter, San Diego, CA, USA), and the percentage of living cells, apoptotic cells, and necrotic cells was calculated using Kaluza software (Beckman Coulter).

### 2.8. Statistical Analysis

All data are expressed as the mean ± standard deviation (STD), and a *p* < 0.05 was regarded as significant. A two-tailed unpaired Student's *t*-test or analysis of variance (ANOVA) was used to compare two or more than three groups, respectively. ANOVA with a post hoc Scheffe's test was used to evaluate the statistical significance of differences between groups.

## 3. Results

### 3.1. miR-302 Binds to the Mcl-1 mRNA 3′UTR

**Figure 1:**
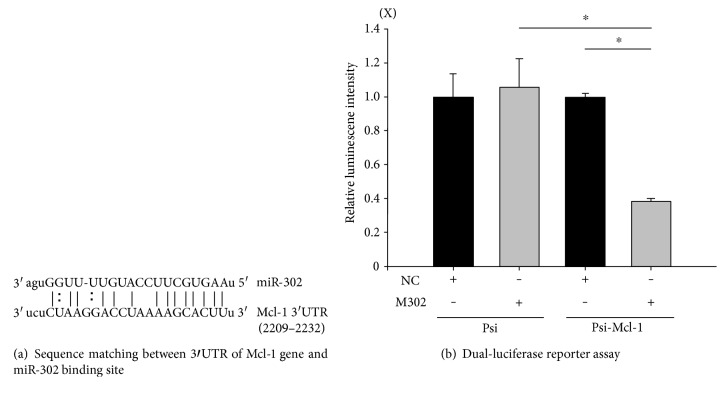
miR-302 regulates the transcriptional activity of *Mcl-1* by targeting its 3′UTR. (a) The 3′UTR sequence (2209–2232) of *Mcl-1* contains a miR-302a-binding site as predicted by miRanda (http://microRNA.org). (b) Mcl-1 is a miR-302 target based on dual-luciferase reporter results. Luciferase activity was evaluated in H9c2 cells transfected with the PsiCheck2 vector only or with PsiCheck2 vectors containing the Mcl-1 3′UTR with nonsense control (NC) or a miR-302 mimic (m302). Data are presented as the mean ± STD of six experiments. ^∗^*p* < 0.05.

To determine whether miR-302 binds to Mcl-1 mRNA, we utilized a reporter gene (luciferase) conjugated to the Mcl-1 3′UTR inserted into a PsiCheck2 vector. We predicted that upon miR-302 binding to the Mcl-1 3′UTR (Figure 1(b), mirSVR score: −1.094; http://www.microrna.org/), reporter-gene expression would decrease. miRs exhibiting a mirSVR score of ≤−1.0 represent the top 7% of predictions and exhibit a >35% probability of having a (Z-transformed) log expression change of at least −1 concerning downregulation of the predicted gene [24]. Cells were transfected with the following vector combinations: (1) luciferase vector not containing the Mcl-1 3′UTR (PsiCheck2), (2) luciferase vector containing the Mcl-1 3′UTR (psi-Mcl-1) and the NC vector, (3) PsiCheck2 and the vector containing m302, (4) psi-Mcl-1 and the NC vector, or (5) psi-Mcl-1 and the vector containing m302. Transfection with psi-Mcl-1 and m302, but not psi-Mcl-1 and NC, PsiCheck2 and m302, or PsiCheck2 and NC decreased luciferase activity (Figure 1(b)). These results indicated that miR-302 binds to the target sequence in the Mcl-1 3′UTR.

### 3.2. Transfection of the miR-302 Mimic and Antagomir Upregulates and Downregulates miR-302 Expression, Respectively, in an H/R Injury Model

**Figure 2:**
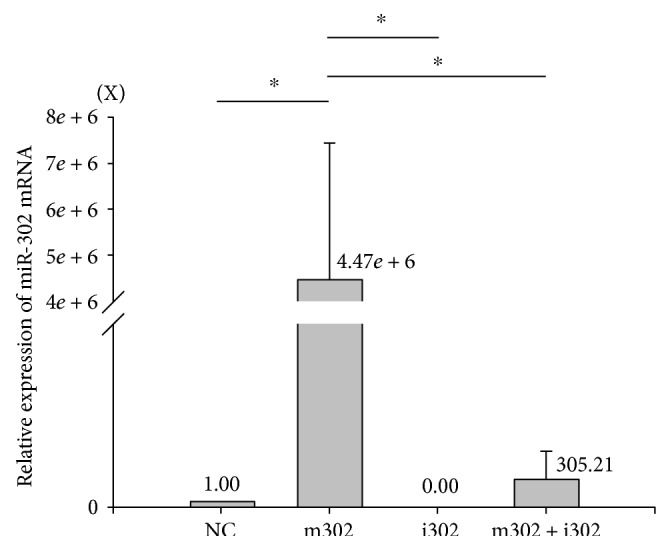
Assessment of miR302 expression and transfection efficiency as determined by real-time qPCR analysis in H9c2 cells exposed to H/R injury. Data are presented as the mean ± STD of six experiments. ^∗^*p* < 0.05. NC: nonsense control; m302: miR-302 mimic; i302: miR-302 antagomir.

We examined the effects of H/R injury on miR-302 expression in H9c2 cardiomyocytes following transfection with NC, m302, and i302. Results of qPCR analysis (Figure 2) showed that miR-302 expression increased significantly in H9c2 cells transfected with m302 or i302 as compared with cells transfected with NC following H/R injury, respectively.

### 3.3. miR-302 Regulates Mcl-1 mRNA and Protein Levels in H/R-Injured Cardiomyocytes

**Figure 3:**
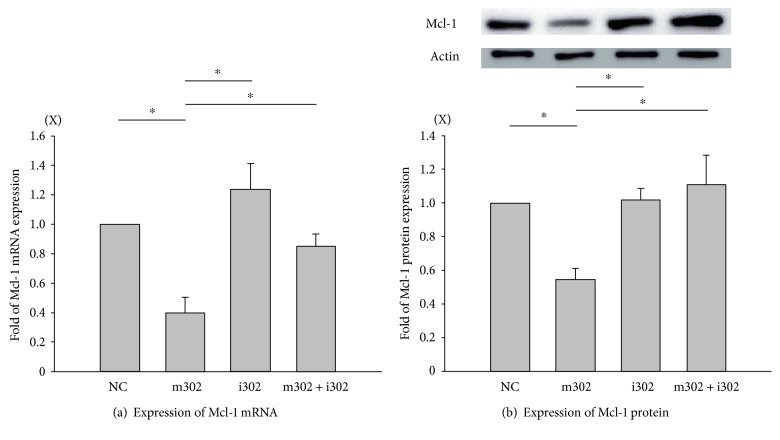
H9c2 cardiomyocytes subjected to H/R injury in the presence of different concentrations of nonsense control (NC), miR-302 mimic (m302), and/or miR-302 antagomir (i302). Mcl-1 expression was assessed by (a) real-time qPCR and (b) western blot analysis. *β*-actin was used as an internal control. Data are presented as the mean ± STD of six experiments. ^∗^*p* < 0.05 between two groups.

qPCR analysis showed that transfection with m302 or i302 (compared with transfection with NC) significantly decreased and increased Mcl-l mRNA levels in H9c2 cardiomyocytes following H/R injury, respectively, as compared with levels observed in NC-transfected controls (Figure 3(a)). Mcl-1 mRNA levels in H9c2 cardiomyocytes cotransfected with m302 and i302 prior to H/R injury were significantly higher and lower, respectively, than those observed in cells transfected with either m302 or i302 alone. Western blot analyses showed that patterns of Mcl-1 protein expression following H/R injury in H9c2 cardiomyocytes transfected with either NC, m302, or i302 matched levels of Mcl-1 mRNA expression (Figure 3(b)); however, Mcl-1 mRNA levels were lower in cells cotransfected with m302 and i302 as compared with levels in cells transfected with i302 alone; although, the difference was not significant.

### 3.4. miR-302 Induces Cardiomyocyte Injury

**Figure 4:**
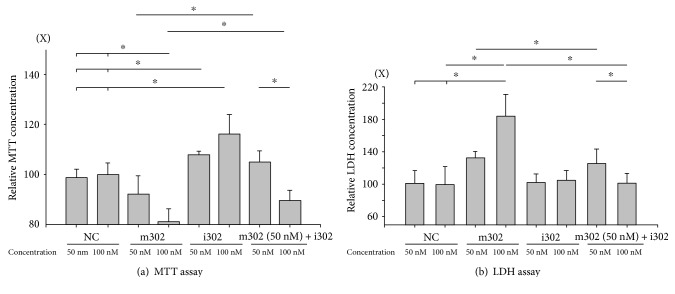
H9c2 cardiomyocytes were subjected to H/R injury in the presence of different concentrations of miR-302 mimic (m302) and/or miR-302 antagomir (i302). Relative viability was assessed by (a) MTT and (b) LDH assays. Data are presented as the mean ± STD of six experiments. ^∗^*p* < 0.05 compared with the group transfected with nonsense control (NC).

To examine the possible damage attributable to miR-302 expression during H/R injury, H9c2 cardiomyocyte viability was evaluated by MTT and LDH assays. We observed that LDH levels increased significantly in cells transfected with m302 (50 nM: 132.7 ± 7.9%; or 100 nM: 184.5 ± 26.4%) versus NC (50 nM: 103.2 ± 12.9%; or 100 nM: 100 ± 21.9%; *p* < 0.05 for both groups (*n* = 6)) (Figure 4(b)). Our results showed that LDH levels increased along with higher concentrations of transfected m302 (*p* = 0.047; *n* = 6). Additionally, the amount of LDH released from cardiomyocytes following H/R injury was reduced by decreasing the expression of miR-302 following i302 transfection and by elevating Mcl-1 expression. Cotransfection of m302 (50 nM) and i320 (50 nM and 100 nM) also significantly decreased the amount of LDH released (50 nM m302: 125.3 ± 18.2%; and 50 nM i302: 101.0 ± 12.8%) during H/R injury, and cotransfection with the higher concentration of i302 decreased LDH release to an even greater extent (*p* = 0.048).

However, during H/R injury, m302 transfection (50 nM: 92.3 ± 7.2%; and 100 nM: 81.2 ± 5.2%) decreased cell viability, whereas i302 transfection increased cell viability (50 nM: 107.9 ± 1.5%; and 100 nM: 116.2 ± 7.8%) according to MTT assay results (Figure 4(a)). The increases and decreases in viability were more pronounced in cells transfected with higher concentrations (50 nM versus 100 nM) of m302 (*p* = 0.02) or i302 (*p* = 0.03). These results indicated that cotransfection with i302 and m302 increased cell viability as compared with levels observed following m302 transfection alone.

### 3.5. i302 Transfection Decreases Cell Apoptosis and Necrosis in H/R-Injured Cardiomyocytes

**Figure 5:**
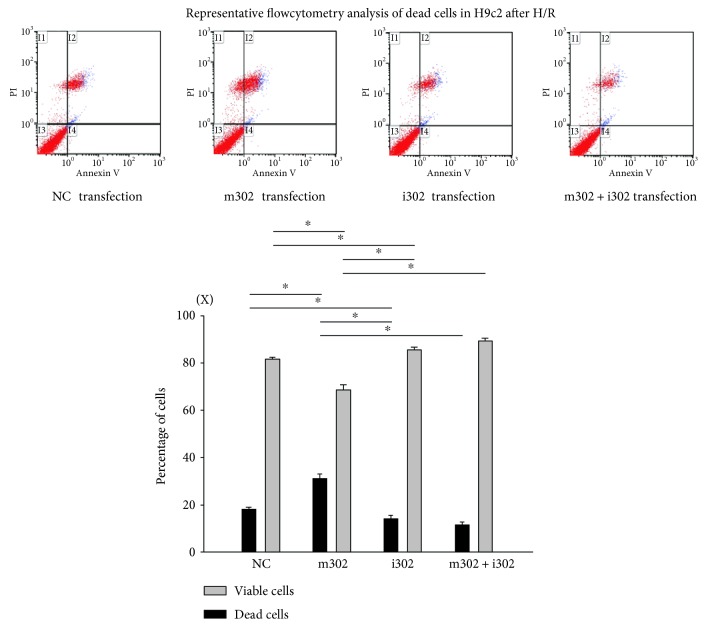
Flow cytometry analysis of cells subjected to H/R injury and transfection with nonsense control (NC), miR-302 mimic (m302; 100 nM), miR-302 antagomir (i302; 100 nM), or m302 + i302 (100 nM). The percentage of viable and apoptotic cardiomyocytes was analyzed in different groups. Data are presented as the mean ± STD of six experiments. ^∗^*p* < 0.05 between two groups.

Levels of apoptosis and necrosis can be elevated by stress. Flow cytometry with Annexin V and PI staining were used to determine levels of cardiomyocyte apoptosis or necrosis during H/R injury and whether changes in apoptosis and necrosis are aggravated or mitigated by miR-302 expression. Cells not stained by either Annexin V or PI were considered healthy. The number of PI-stained cells (necrotic) and PI + Annexin V costained cells (late apoptotic) was calculated as a percentage of the total number of cardiomyocyte nuclei (Figure 5). Following H/R injury, the percentage of apoptotic H9c2 cardiomyocytes was significantly higher following m302 transfection (30.4 ± 2.2%) as compared with that observed following NC transfection (17.5 ± 0.7%; *p* = 0.0012). Additionally, the percentage of apoptotic H9c2 cardiomyocytes was significantly lower following i302 transfection (13.2 ± 1.0%; *p* = 0.014) and i302 and m302 cotransfection (9.1 ± 0.8%; *p* = 0.0003) as compared with percentages accompanying NC transfection.

### 3.6. miR-302 Regulates the Expression and Activation of Apoptosis-Related Proteins

**Figure 6:**
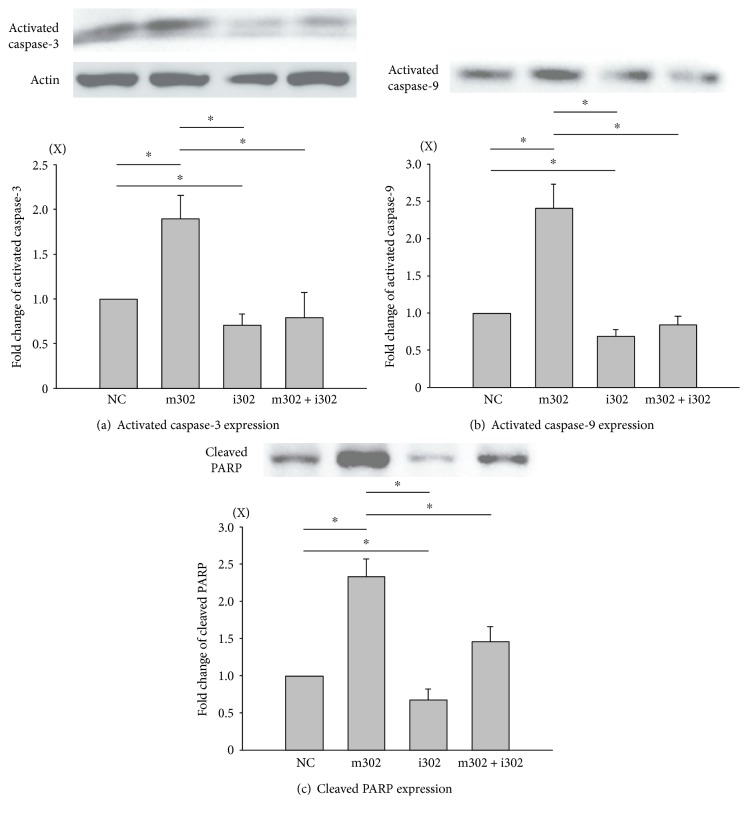
H9c2 cardiomyocytes subjected to H/R injury in the presence of different concentrations of nonsense control (NC), miR-302 mimic (m302), and/or miR-302 antagomir (i302). (a) Activated caspase-3, (b) activated caspase-9, and (c) cleaved PARP in different groups were analyzed by western blot. *Β*-actin was used as an internal control. Data are presented as the mean ± STD of six analyses. ^∗^*p* < 0.05 between two groups.

To determine whether the underlying mechanism of apoptotic suppression involves inhibition of miR-302 expression, levels of apoptosis-related proteins were evaluated by western blot analysis in cells transfected with NC, m302, i302, or m302 + i302 (Figure 6). Consistent with previous data, H9c2 cells transfected with m302 and exhibiting elevated miR-302 expression and reduced Mcl-1 expression also exhibited significantly increased levels of activated caspase-3 and -9 (both *p* < 0.05), which significantly increased the expression of cleaved PARP (both *p* < 0.05). However, cells transfected with i302 or cotransfected with m302 + i302 exhibited significantly reduced levels of activated caspase-3 and -9 and cleaved PARP.

## 4. Discussion

miRs are critical regulators of cell function [25] and influence a myriad of cell characteristics, including stem cell reprogramming, cardiac differentiation, and hypertrophy [5]. Successful manipulation of miRs can improve outcomes of cardiovascular disease and constitutes a new cardiovascular therapeutic strategy [26, 27]. In this study, we showed that decreasing miR-302 expression by transfection of a miR-302 antagonist elevated the expression of the antiapoptotic protein Mcl-1, decreased the activation of caspase-3 and -9, and reduced cardiomyocyte injury and death during H/R injury.

The antiapoptotic Mcl-1 protein is a member of the Bcl-2 family, a suppressor of apoptosis, and enhances cell viability during H/R [28]. Here, we confirmed that the Mcl-1 3′UTR is a miR-302 target. miR-302 plays a role in preventing cell death resulting from oxidant-induced damage [4] and affects the reprogramming of stem cells and somatic cells [29]. Additionally, miR-302-transfected cells can be used to repair heart damage [30], and another study found that miR-302 can inhibit the expression of transformation-related genes and exhibit a deprogramming effect on glioblastoma cells, implying that miR-302 could also be a cancer therapeutic target [31]. Regarding the effects of miR-302 on cell proliferation and reprogramming, Kuppusamy et al. [5] reported that miR-302 plays an important role in cardiomyocyte differentiation and maturation, especially after myocardial infarction [5]. However, the possible differential effects associated with miR-302 on cardiomyocytes under H/R injury had not been previously studied. According to our hypothesis, miR-302 downregulation would result in elevation of Mcl-1 expression and reductions in cardiomyocyte death. We found that H/R-induced cell death was potentiated by miR-302 overexpression following m302 transfection, whereas decreased miR-302 expression via its antagomir (i302) protected cells from H/R injury. Moreover, cotransfection of i302 with m302 mitigated H/R-induced cell injury. Our results showed for the first time that elevated miR-302 expression was harmful to H/R-injured cardiomyocytes.

Additionally, we confirmed that miR-302 binds to the Mcl-1 3′UTR and decreases Mcl-1 mRNA and protein levels according to luciferase reporter results. Mcl-1 is capable of inhibiting the activation of proapoptotic proteins, such as Bax, Bak, or Bid, and the release of cytochrome *c* from mitochondria, thereby preventing apoptosis during H/R [19]. In H9c2 cells, m302 transfection elevated miR-302 expression, resulting in decreased Mcl-1 mRNA and protein levels during H/R, whereas i302 transfection in the presence or absence of m302 transfection attenuated H/R-induced miR-302 expression, rescued Mcl-1 mRNA and protein levels, and decreased cell injury. These results indicated that Mcl-1 expression was inhibited by miR-302 during H/R injury. Analysis of the percentage of cells undergoing apoptosis by Annexin V/PI double staining revealed that m302 transfection increased rates of cell death following H/R, whereas cotransfection with i302 significantly decreased the percentage of apoptotic cells. Therefore, inhibition of miR-302 expression decreased cytotoxicity, increased viability, and decreased apoptosis during H/R by promoting increased Mcl-1 protein levels.

Our results showed that the effects of m302 could be reversed by i302 cotransfection. miR-302 levels following m302 transfection were elevated as compared with those observed in other groups (Figure 2). Additionally, Mcl-1 mRNA levels were significantly decreased only in the m302-transfected group, indicating that high levels of miR-302 were capable of attenuating Mcl-1 levels. This finding verified our hypothesis that miR-302 was induced in cardiomyocyte H/R injury and subsequently inhibited the expression of Mcl-1 mRNA and protein.

Cardiomyocyte apoptosis is a key cellular event in cardiac ischemia/reperfusion injury [32]. Changes in Mcl-1 expression, as well as that of other key elements of the apoptosis pathway, such as phosphoinositide 3-kinase, Bcl-2, heat-shock protein 60, heat-shock protein 70, PDCD4, and Fas ligand, alter cellular response to ischemia-reperfusion injury [33]. Mcl-1 is involved in cardiomyocyte loss and contributes to a variety of cardiac pathologies, including heart failure and ischemia/reperfusion injury [34, 35]. Additionally, Mcl-1 is known to function as an upstream antagonist of the intrinsic death activators caspase-3 and -9, thereby inhibiting apoptosis while also being essential for maintaining cardiac mitochondrial homeostasis and inducing autophagy in the heart [34]. Wang et al. [35] found that Mcl-1 ablation results in a loss of cardiac contractility, followed by fatal cardiomyopathy [35]. Similar to our findings concerning miR-302, Zou et al. [8] reported that miR-153 targets Mcl-1 to modulate apoptosis and autophagy, thereby regulating the survival of cardiomyocytes during oxidative stress [8]. Here, miR-302 overexpression or knockdown alone was shown to decrease and increase Mcl-1 protein levels, respectively.

Restoration of blood flow or reoxygenation produces free radicals that harm cells and tissues. In developing countries, myocardial infarction is a primary cause of death, because therapies to restore blood circulation are not always available. Therefore, maintenance of cardiomyocyte function to improve heart contraction is a potential therapeutic approach. According to recent studies, miRs regulate cells or tissues exposed to myocardial infarction or ischemia, with reports finding that miR-21 protects hearts from ischemic injury via PDCD4 expression [2], miR-361 transfected into rat hearts causes mitochondrial fission and myocardial ischemic injury [36], and miR-30b protects cardiomyocytes by inhibiting cyclophilin D [3]. Embryonic stem cells express high levels of the miR-302/367 cluster, with this expression regulated by the binding of transcription factors, such as octamer-binding transcription factor 3/4, SRY-box 2, and Nanog, to miR-302/367-cluster promoter regions. The miR-302/367 cluster subsequently increases stem cell self-renewal capacity, pluripotency, and maintenance of stemness [37]. miR-302 inhibits differentiation by inhibiting the differentiation factors Lefty1 and Lefty2 to maintain embryonic stem cell proliferation [37]. Furthermore, increases in hydrogen peroxide in quiescent fibroblasts lead to miR-302-mediated regulation of the cell cycle regulatory factor AT-rich interaction domain 4A and chemokine (C-C motif) ligand-5 mRNA levels [38]. Other cell-signaling pathways, such those related to bone morphogenetic protein signaling and the tumor necrosis factor-*β*/Nodal/Smad-2/3 pathway, are also regulated by the miR-302/367 cluster [39]. Here, our data showed that miR-302 increased cardiomyocyte apoptosis and regulated Mcl-1 mRNA and protein levels, resulting in elevated levels of Mcl-1 preventing cell death or tissue damage and decreased levels promoting apoptosis. Because miR-302 transfection preserves stem cell stemness, cells transfected with miR-302 and transplanted into the heart have been used to explore their ability to promote cell proliferation and the repair of cardiomyocytes damaged by myocardial infection. Similarly, we examined the effect of miR-302 transfection on H/R-injured cardiomyocytes, revealing that elevated miR-302 levels were detrimental to cells, whereas decreased miR-302 levels were beneficial according to an H/R model. These findings suggested that reducing miR-302 levels in cardiomyocytes during H/R might constitute an effective therapeutic intervention.

## 5. Conclusions

We found that miR-302 mediates H/R-induced cardiomyocyte death by regulating Mcl-1 expression. Therefore, miR-302 inhibition might offer an effective therapeutic strategy for the treatment of myocardial infarction or ischemia-related diseases.
